# Why measurements do (not) work: the human factor

**DOI:** 10.1186/cc13339

**Published:** 2014-03-17

**Authors:** A Rameau, NA Bruins, P Egbers, MA Kuiper, EC Boerma

**Affiliations:** 1 Medical Center Leeuwarden, the Netherlands

## Introduction

Passive leg raising (PLR) has been suggested as a simple diagnostic tool to guide fluid administration in critically ill patients [1]. Although the basic principles of PLR have been well studied, little is known about the impact of the introduction of this technique in daily clinical practice. The aim of the study was to describe the changes in fluid balance of ICU patients before and after the introduction of PLR, and to determine the compliance of medical personnel with a PLR- driven protocol of fluid administration.

## Methods

In this single-centre prospective study, mixed ICU patients equipped with a PiCCO system received fluid therapy on the basis of PLR test results in the first 48 hours of treatment, after careful introduction of a new PLR-driven protocol. Exclusion criteria were increased abdominal pressure, fracture of leg or pelvis, deep vein thrombosis, head trauma and pregnancy. The control group existed of patients admitted to the ICU 1 year prior to the introduction of the protocol.

## Results

**Figure 1:**
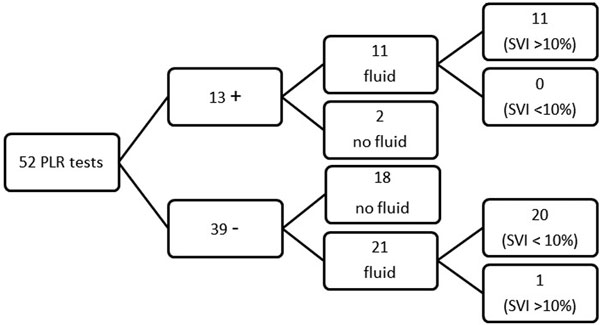
**SVI, stroke volume index; -, negative PLR; +, positive PLR; fluid 250 cm^3 ^RL solution**.

We included 21 patients in each group. There was no significant differences in the fluid balance between the control and study group after 24 hours (5.0 ± 2.9 l vs. 3.6 ± 2.7 l, *P *= 0.11) and 48 hours (5.7 ± 3.5 l vs. 4.8 ± 3.7 l, *P *= 0.39). However, compliance with the protocol was poor (56%). After 2/11 positive tests, fluid was not administered; and after 21/39 tests, fluid was administered despite a negative test result (Figure 1).

## Conclusion

The implementation of a PLR-driven protocol of fluid administration did not change mean fluid balances after the first 48 hours. Noncompliance with the protocol was an important confounder.
